# Whole exome sequencing of Rett syndrome-like patients reveals the mutational diversity of the clinical phenotype

**DOI:** 10.1007/s00439-016-1721-3

**Published:** 2016-08-19

**Authors:** Mario Lucariello, Enrique Vidal, Silvia Vidal, Mauricio Saez, Laura Roa, Dori Huertas, Mercè Pineda, Esther Dalfó, Joaquin Dopazo, Paola Jurado, Judith Armstrong, Manel Esteller

**Affiliations:** 1 Cancer Epigenetics and Biology Program (PEBC), Bellvitge Biomedical Research Institute (IDIBELL), L’Hospitalet, 08908 Barcelona, Catalonia Spain; 2 Servei de Medicina Genètica i Molecular, Institut de Recerca Pediàtrica Hospital Sant Joan de Déu, Esplugues De Llobregat, Catalonia Spain; 3 Fundació Hospital Sant Joan de Déu (HSJD), Barcelona, Catalonia Spain; 4 Genetics Department, Bellvitge Biomedical Research Institute (IDIBELL), Barcelona, Catalonia Spain; 5 Computational Genomics Department, Centro de Investigación Príncipe Felipe (CIPF), 46012 Valencia, Spain; 6 Bioinformatics of Rare Diseases (BIER), CIBER de Enfermedades Raras (CIBERER), Valencia, Spain; 7 Functional Genomics Node (INB) at CIPF, 46012 Valencia, Spain; 8 CIBER Enfermedades Raras, Barcelona, Catalonia Spain; 9 Department of Physiological Sciences, School of Medicine and Health Sciences, University of Barcelona, Barcelona, Catalonia Spain; 10 Institucio Catalana de Recerca i Estudis Avançats (ICREA), Barcelona, Catalonia Spain; 11 Department of Neurology, Hospital Sant Joan de Déu (HSJD), Barcelona, Catalonia Spain

## Abstract

Classical Rett syndrome (RTT) is a neurodevelopmental disorder where most of cases carry *MECP2* mutations. Atypical RTT variants involve mutations in *CDKL5* and *FOXG1*. However, a subset of RTT patients remains that do not carry any mutation in the described genes. Whole exome sequencing was carried out in a cohort of 21 female probands with clinical features overlapping with those of RTT, but without mutations in the customarily studied genes. Candidates were functionally validated by assessing the appearance of a neurological phenotype in *Caenorhabditis elegans* upon disruption of the corresponding ortholog gene. We detected pathogenic variants that accounted for the RTT-like phenotype in 14 (66.6 %) patients. Five patients were carriers of mutations in genes already known to be associated with other syndromic neurodevelopmental disorders. We determined that the other patients harbored mutations in genes that have not previously been linked to RTT or other neurodevelopmental syndromes, such as the ankyrin repeat containing protein *ANKRD31* or the neuronal acetylcholine receptor subunit alpha-5 (CHRNA5). Furthermore, worm assays demonstrated that mutations in the studied candidate genes caused locomotion defects. Our findings indicate that mutations in a variety of genes contribute to the development of RTT-like phenotypes.

**Electronic supplementary material:**

The online version of this article (doi:10.1007/s00439-016-1721-3) contains supplementary material, which is available to authorized users.

## Introduction

Rett syndrome (RTT, MIM 312750) is a postnatal progressive neurodevelopmental disorder (NDD), originally described in the 1960s by Andreas Rett (Rett 1966), that most frequently manifests itself in girls during early childhood, with an incidence of approximately 1 in 10,000 live births (Chahrour and Zoghbi 2007). RTT patients are asymptomatic during the first 6–18 months of life, but gradually develop severe motor, cognitive, and behavioral abnormalities that persist for life. It is the second most common cause of intellectual disability in females after Down’s syndrome (Chahrour and Zoghbi 2007). Around 90 % of the cases are explained by more than 800 reported mutations in the methyl CpG-binding protein 2 gene (*MECP2*) (RettBASE: MECP2 Variation Database) (Christodoulou et al. 2003), which is located in the X chromosome and which causes most of the classical or typical forms of RTT (Chahrour and Zoghbi 2007), and it was originally identified as encoding a protein that binds to methylated DNA (Lewis et al. 1992). Individuals affected by atypical or variant RTT present with many of the clinical features of RTT, but do not necessarily have all of the classic characteristics of the disorder (Neul et al. 2010). Approximately 8 % of classic RTT and 42 % of variant RTT patients are *MECP2* mutation-negative (Monros et al. 2001; Percy 2008). Some of the latter group have mutations in other genes, such as that of the cyclin-dependent kinase-like 5 (*CDKL5*), which is described in individuals with an early seizure onset variant of RTT (Kalscheuer et al. 2003) or the forkhead box G1 (*FOXG1*), which is responsible for the congenital variant of RTT (Ariani et al. 2008). However, there remains a subset of patients with a clinical diagnosis of RTT who are mutation-negative for all the aforementioned genes. Next generation sequencing (NGS) has emerged as a potentially powerful tool for the study of such genetic diseases (Zhu et al. 2015).

Herein, we report the use of a family based exome sequencing approach in a cohort of 20 families with clinical features of RTT, but without mutations in the usually studied genes. We establish the neurological relevance of the newly identified candidate genes by assessing them in *Caenorhabditis elegans* model.

## Materials and methods

### Patient samples

A cohort of 19 Spanish parent–child trios and one family with two affected daughters who exhibited clinical features associated with RTT were recruited at Sant Joan de Deu Hospital in Barcelona, Catalonia, Spain. These patients had been diagnosed on the basis of the usual clinical parameters (Monros et al. 2001), and according to the recently revised RettSearch International Consortium criteria and nomenclature (Neul et al. 2010), but were found to be mutation-negative for *MECP2*, *CDKL5* and *FOXG1* in the original single-gene screening. The parents were clinically evaluated and it was not observed any evidence of intellectual disability. Genomic DNA from these patients was extracted from peripheral blood leukocytes using standard techniques, and analyzed by exome sequencing at the Cancer Epigenetics and Biology Program (PEBC) in Barcelona, Catalonia, Spain. Ethical approval for the molecular genetic studies was obtained from each institutional review board.

### Whole exome sequencing and Sanger validation

Coding regions were captured using the TruSeq DNA Sample Preparation and Exome Enrichment Kit (Illumina, San Diego, California). Paired-end 100 × 2 sequences were sequenced with the Illumina HiScan SQ system at the National Center for Genomic Analysis in Barcelona. We also included the exome sequencing data of an *MECP2*, a *CDKL5* and a *JMJD1C* (Sáez et al. 2016) RTT-associated family for data processing to improve the de novo single nucleotide variant calling. The complete exome sequencing data of all the studied samples are available from the Sequence Read Archive (http://www.ncbi.nlm.nih.gov/sra) with the ID: SRP073424 (private link for the reviewer until publication: http://www.ncbi.nlm.nih.gov/sra/SRP073424). The overall coverage statistics for each individual of the families, considering the regions captures using Exome Enrichment Kit, and number of reads in the position of the variation is shown in Supplementary Table 1. The identified variants were validated by Sanger sequencing using a BigDye^®^ Terminator v3.1 Cycle Sequencing Kit in an Applied Biosystems 3730/DNA Analyzer. The raw data were analyzed with Codon Code Aligner Software. The primers used for Sanger sequencing are shown in Supplementary Table 2.

### *Caenorhabditis elegans* handling

The techniques used for the culture of *Caenorhabditis elegans* were essentially as described (Brenner 1974). The worms were backcrossed at least three times to avoid background mutations. The behavior of three sets of ten animals was independently assessed in locomotion assays without food that were performed at 20 °C, as previously described (Sawin et al. 2000).

## Results

### Clinical criteria for selecting RTT trios

The 21 patients (derived from the 20 families studied) included in this study fulfilled the recently revised clinical criteria for the diagnosis of RTT following the usual clinical parameters (Monros et al. 2001), and the RettSearch International Consortium criteria and nomenclature (Neul et al. 2010). Specifically, all patients presented stereotypic hand movements, 90.5 % of them (19/21) showed microcephaly and also presented onset of the first signs of the disease before the age of 12 months. 66.7 % of patients (14/21) acquired motor skills, while a further seven (33.3 %), who had a more severe phenotype, never walked. Language skills were progressively lost in 28.6 % of the patients and 71.4 % of them (15/21) never acquired them. Additionally, important episodes of epilepsy were experienced by 81.0 % of the patients (17/21), and 57.1 % of them (12/21) manifested apneas and/or hyperventilation.

### Bioinformatic process for filtering and selecting pathogenic variants

Before their inclusion in this study, patients underwent an extensive clinical and genetic work-up to detect genetic alterations in *MECP2*, *CDKL5*, and *FOXG1*. However, no molecular diagnosis could be established. We performed whole exome sequencing (WES) on the 61 individuals (20 pairs of healthy parents and 21 affected daughters) separately by subjecting whole blood derived genomic DNA to exome enrichment and sequencing. We focused our analysis on de novo single nucleotide variants (SNVs) due to their known relevance in autism and mental retardation-related diseases (Vissers et al. 2010). On average, WES gave rise to 419,045 variants, including SNVs and indels, of which 19,951 non-synonymous variants per family (4.7 %) were predicted to have a functional impact on the genomic sequence. To select variants that had not previously been described in the healthy population, we filtered out the variants with an allele frequency of 1 % or higher (the classic definition of a polymorphism) formerly observed in the Single Nucleotide Polymorphism database (dbSNP) and the 1000 Genomes Pilot Project data. Afterwards, to focus on de novo inheritance, patients’ variants were filtered first against variants found in their own parents and then against a pool of controls comprising all the healthy parents included in the study. Following this process, we achieved an average of 106 SNVs per family, which corresponded to 81 mutated genes per family. De novo candidate variants were selected on the basis of the quality of the alignments, damage score predictors and the conservation level of each of the genes during evolution. The complete exome sequencing data of all the studied samples are available from the Sequence Read Archive (http://www.ncbi.nlm.nih.gov/sra).

**Fig. 1:**
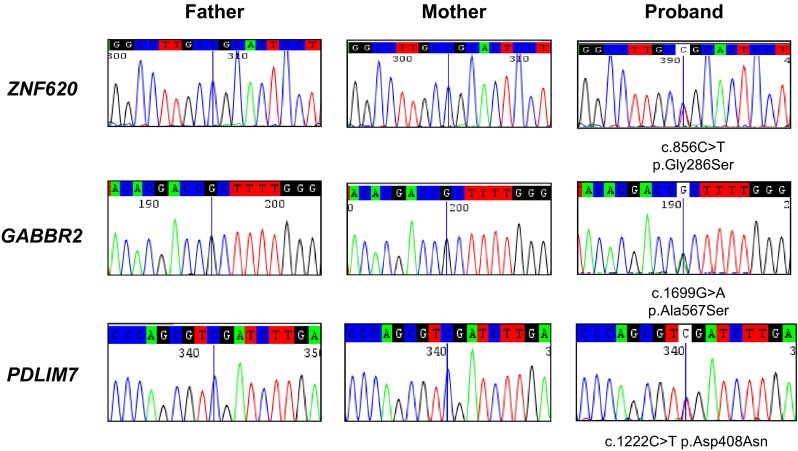
Sanger sequencing validation of the de novo variants identified by exome sequencing. Illustrative examples for ZNF620 (c.856C > T p.Gly286Ser), GABBR2 (c.1699G > A p.Ala567Ser) and PDLIM7 (c.1222C > T p.Asp408Asn) are shown

The global yield of genomic analysis following the bioinformatic process described herein enabled 22 coding de novo mutations to be identified in 66.7 % (14 of 21) of Rett-like patients: 20 SNVs and 2 indels. The identified variants and their de novo status were confirmed by conventional Sanger sequencing. Illustrative samples are shown in Fig. 1. Interestingly, in seven (33.3 %) of the studied RTT probands, exome sequencing did not detect any genetic change relative to their respective parents. The clinical characteristics of these seven patients without obvious pathogenic variants are summarized in Table 1. In one of the families, there were two affected children, and an analysis of potentially relevant recessive variants was performed. For the recessive analysis, and following the same criteria to define a variant as deleterious, we selected the variants with homozygous recessive genotype, and then at the gene level, we also selected the genes presenting more than one heterozygotic variant in the same gene (compound heterozygosity). We did not find any candidate gene consistent with the phenotype of the family with the two affected sisters.

**Table 1:** Clinical summary of patients without exome candidates

Proband	Age (years)	Onset of signs	Microcephaly	Sitting alone	Ambulation	Respiratory function	Epilepsy	Hand use	Stereotypies	Language	Total score
1	15	1	1	1	1	0	1	1	1	1	8
2	28	3	1	1	0	1	2	2	3	2	15
5.1	7	3	1	3	4	1	0	3	2	2	19
5.2	5	3	1	1	2	0	0	2	3	2	14
7	16	2	1	0	0	0	2	1	2	1	9
15	8	3	0	1	2	0	2	1	1	1	10
18	3.5	3	1	1	1	0	0	1	3	0	10

Clinical scores of our series of patients according to Pineda scale. Severity classification ranges from 0 to 4 as follows: age of onset of first signs (1: >24 months; 2: 12–24 months; 3: 0–12 months), microcephaly (0: absent; 1: present), sitting alone (1: acquired < 8 months; 2: seat and mantains; 3: seat and lost), ambulation (0: acquired < 18 months, 1: acquired < 30 months; 2: acquired > 30 months; 3: lost acquisition; 4: never acquired), respiratory function (0: no dysfunction; 1: hyperventilation and/or apnea), epilepsy (0: absent; 1: present and controlled; 2: uncontrolled or early epilepsy), hands use (0: acquired and conserved; 1: acquired and partially conserved; 2: acquired and lost; 3: never acquired), onset of stereotypies (1: > 10 years, 2: > 36 months; 3: 18-36 months) and languages (0: preserved and propositive; 1: lost; 2: never acquired). The total score is the sum of the scores of each clinical feature

### Variants in genes previously associated with neurodevelopmental disorders

Of the 22 identified coding de novo mutations in the assessed RTT-like patients, five (22.7 %) occurred in genes previously associated with neurodevelopmental disorders that presented a clinicopathological phenotype with features coinciding with those of Rett syndrome (Table 2). In particular, we identified four mutations in genes such as *HCN1* (Nava et al. 2014) and *GRIN2B* (Endele et al. 2010; Lemke et al. 2014), which are associated with early infantile epileptic encephalopathy; *SLC6A1*, which is associated with epilepsy and myoclonic-atonic seizures (Carvill et al. 2015); *TCF4*, which is associated with Pitt–Hopkins syndrome (Sweatt 2013); and *SCN1A*, which is associated with Dravet syndrome (Brunklaus and Zuberi 2014) (Table 2). The clinical characteristics of these five patients with variants in genes previously associated with neurodevelopmental phenotypes are summarized in Table 3. A comparison of the clinical features of our RTT-like patients, where we have identified mutations in candidate genes previously associated with other neurodevelopmental disorders, with those observed for these diseases is summarized in Table 4.

**Table 2:** List of patients with variants found in genes previously associated with neurodevelopmental phenotypes

Proband	Gene	Protein	NM number	Variant: genomic coordinates	cDNA change	Protein change	Gene-disease association
4	*HCN1*	Hyperpolarization Activated Cyclic Nucleotide Gated Potassium Channel 1	NM_021072.3	5:45396665	c.1159G > T	p.Ala387Ser	Early infantile epileptic encephalopathy 24
8	*SCN1A*	Sodium Channel Protein Type I Subunit Alpha	NM_001165963.1	2:166866266	c.3965C > G	p.Arg1322Thr	Dravet syndrome
10	*TCF4*	Transcription Factor 4	NM_001243236.1	18:52901827	c.958delC	p.Gln320Ser_fs8X	Pitt–Hopkins syndrome
11	*GRIN2B*	Glutamate receptor ionotropic, NMDA 2B	NM_000834.3	12:13764782	c.1657C > A	p.Pro553Thr	Autosomal Dominant Mental Retardation 6; Early infantile epileptic encephalopathy 27
17	*SLC6A1*	Solute Carrier Family 6 Member 1	NM_003042.3	3:11067528	c.919G > A	p.Gly307Arg	Myoclonic-atonic epilepsy and schizophrenia

**Table 3:** Clinical summary of the patients with variants in genes previously associated with neurodevelopmental phenotypes

Proband	Gene variant	Age (years)	Onset of the signs	Microcephaly	Sitting alone	Ambulation	Respiratory function	Epilepsy	Hand use	Stereotypies	Language	Total score
4	*HCN1*	24	3	1	1	3	1	2	3	1	2	16
8	*SCN1A, MGRN1, BTBD9*	7	3	1	1	4	1	2	1	3	2	18
10	*TCF4*	16	3	1	1	2	1	2	1	1	2	14
11	*GRIN2B, SEMA6B*	3	2	0	1	3	0	1	1	1	2	10
17	*SLC6A1*	36	3	0	1	1	0	1	1	1	0	7

Clinical scores of our series of patients according to Pineda scale. Severity classification ranges from 0 to 4 as follows: age of onset of first signs (1: > 24 months; 2: 12–24 months; 3: 0–12 months), microcephaly (0: absent; 1: present), sitting alone (1: acquired < 8 months; 2: seat and mantains; 3: seat and lost), ambulation (0: acquired < 18 months, 1: acquired < 30 months; 2: acquired > 30 months; 3: lost acquisition; 4: never acquired), respiratory function (0: no dysfunction; 1: hyperventilation and/or apnea), epilepsy (0: absent; 1: present and controlled; 2: uncontrolled or early epilepsy), hands use (0: acquired and conserved; 1: acquired and partially conserved; 2: acquired and lost; 3: never acquired), onset of stereotypies (1: > 10 years, 2: > 36 months; 3: 18-36 months) and languages (0: preserved and propositive; 1: lost; 2: never acquired). The total score is the sum of the scores of each clinical feature

**Table 4:** Comparison of the clinical features of RTT-like patients from who we have identified mutations in candidate genes previously associated with other neurodevelopmental disorders with those observed for these diseases

Disease	Rett		Atypical Rett		Pitt–Hopkins		Dravet		EEIE27		MAE		EEIE24	
GENE	*MECP2*		*CDKL5*		*TCF4*		*SCN1A*		*GRIN2B*		*SLC6A1*		*HCN1*	
OMIM/Patient	312,750	6	308,350	16	602,272	10	182,390	8	616,139	11	616,421	17	615,871	4
Onset age	6–18 m	12–24 m	1–3 m	0–12 m	6–12 m	0–12 m	2 days–7 m	0–12 m	0–24 m	12–24 m	0–24 m	0–12 m	0–24 m	0–12 m
Microcephaly	Yes	Yes	Yes	Yes	Yes	Yes	±	Yes	±	No	±	No	NA	Yes
Hypotonia	Yes	Yes	Yes	Yes	Yes	Yes	No	Yes	±	Yes	±	Yes	NA	Yes
Epilepsy	80 %	Yes	Yes	Yes	Yes	Yes	Yes	Yes	±	Yes	Yes	Yes	Yes	Yes
Respiratory dysfunction	80 %	Yes	Yes	Yes	Yes	Yes	Yes	Yes	No	No	No	No	NA	Yes
Expressive language dysfunction	Yes	Yes	Yes	Yes	Yes	Yes	±	Yes	Yes	Yes	No	No	NA	Yes
Preserved use of hands	No	No	±	No	No	No	Yes	No	±	No	±	No	NA	No
Stereotypies	Yes	Yes	Yes	Yes	Yes	Yes	±	Yes	±	Yes	±	Yes	NA	Yes
Inheritance	XL		XL		AD		AD		AD		AD		AD	

*EEIE* epileptic encephalopathy, early infantile, *MAE*, myoclonic-atonic epilepsy, *m* months, *XL* X linkage, *AD* autosomal dominant, *NA* unavailable

### Variants in genes previously not associated with neurodevelopmental disorders

Of the 22 identified coding de novo variants in the RTT-like patients assessed here, 17 (77.3 %) occurred in genes that had not previously been associated with neurodevelopmental disorders (Table 5). However, two of these variants were associated with non-neurodevelopmental disorders: a *BTBD9* variant linked to restless leg syndrome (Kemlink et al. 2009), and an *ATP8B1* SNV associated with familial cholestasis (Klomp et al. 2004), respectively. Interestingly, the *BTBD9* variant was detected in the same patient that carried the *SCN1A* variant associated with Dravet syndrome (Table 2). The other 15 potentially pathogenic variants identified occurred in genes that had not been linked to any genetic disorder of any type. However, there was an enrichment of genes with a potential role in neuronal biology and functionality, such as the gamma-aminobutyric type B receptor subunit 2 (*GABBR2*), the neuronal acetylcholine receptor subunit alpha-5 (*CHRNA5*), the Huntington-associated protein 1 (*HAP1*), the axon guider semaphorin 6B, the ankyrin repeat containing proteins *ANKRD31* and *AGAP6*, and the neuronal voltage-gated calcium channel *CACNA1* (Table 5). Proband 14 was a particularly interesting case in which four potential pathogenic variants were present, affecting zinc finger (*ZNF620*), a nucleolar complex (*NOC3L*), G patch domain (*GPATCH2*) and GRAM domain (*GRAMD1A*)-related proteins (Table 5). The clinical characteristics of these patients with variants in genes previously not associated with neurodevelopmental disorders are summarized in Table 6.

**Table 5:** List of patients with variants in new candidate disease genes

Proband	Gene	Protein	Function	NM number	Variant: genomic coordinates	cDNA change	Protein change	ExAC	SIFT	Polyphen2	PROVEAN	Mutation Taster2	Conservation
3	*AGAP6*	ArfGAP with GTPase domain, ankyrin repeat and PH domain 6	Putative GTPase-activating protein	NM_001077665.2	10:51748528	c.53insC	p.Asp18Ala_fs10X	Not present	NA	NA	B	P	405
8	*MGRN1*	Mahogunin RING Finger Protein 1	E3 ubiquitin-protein ligase	NM_001142290.2	16:4723583	c.880C > T	p.Arg294Cys	0.000077	P	P	P	P	573
8	*BTBD9*	BTB (POZ) Domain-Containing 9	Putative protein–protein interactor	NM_001099272.1	6:38256093	c.1409C > T	p.Ala470Val	Not present	B	P	P	P	512
11	*SEMA6B*	Semaphorin-6B	Role in axon guidance	NM_032108.3	19:4555540	c.508G > A	p.Gly170Ser	Not present	P	P	P	P	510
12	*VASH2*	Vasohibin 2	Angiogenesis inhibitor	NM_001301056.1	1:213161902	c.1044A > C	p.Glu348Asp	Not present	B	B	B	B	473
13	*CHRNA5*	Neuronal acetylcholine receptor subunit alpha-5	Excitator of neuronal activity	NM_000745.3	15:78882481	c.748C > A	p.Pro250Thr	Not present	B	P	P	P	519
14	*ZNF620*	Zinc Finger Protein 620	Transcriptional regulator	NM_175888.3	3:40557941	c.856G > A	p.Gly286Ser	Not present	P	P	P	P	317
14	*GRAMD1A*	GRAM Domain-Containing 1A	Not described	NM_020895.3	19:35506764	c.1106G > A	p.Arg369His	Not present	P	P	P	P	358
14	*NOC3L*	Nucleolar complex protein 3 homolog	Regulator of adipogenesis	NM_022451.10	10:96097586	c.2137G > A	p.Ala713Thr	Not present	B	B	B	B	0
14	*GPATCH2*	G patch domain-containing protein 2	Regulator of cell proliferation	NM_018040.3	1:217784371	c.878G > A	p.Gly293Asp	Not present	B	P	P	P	304
19	*GABBR2*	Gamma-aminobutyric acid type B receptor subunit 2	Inhibitor of neuronal activity	NM_005458.7	9:101133817	c.1699G > A	p.Ala567Thr	Not present	P	P	P	P	412
19	*ATP8B1*	Phospholipid-transporting ATPase IC	Aminophospholipid translocator	NM_005603.4	18:55328507	c.2606C > T	p.Thr869Ile	Not present	P	P	P	P	361
20	*HAP1*	Huntingtin-Associated Protein 1	Vesicular transporter	NM_177977.2	17:39890655	c.232G > A	p.Ala78Thre	Not present	P	B	B	B	0
21	*PDLIM7*	PDZ and LIM domain protein 7	Scaffold protein	NM_005451.4	5:176910933	c.1222G > A	p.Asp408Asn	Not present	P	P	B	P	515
21	*SRRM3*	Serine/Arginine Repetitive Matrix 3	Splicing activator	NM_001291831.1	7:75890878	c.655C > G	p.Ser218Cys	Not present	P	P	P	P	491
22	*ANKRD31*	Ankyrin Repeat Domain 31	Not described	NM_001164443.1	5:74518166	c.196A > T	p.Ile66Phe	Not present	P	P	B	B	401
23	*CACNA1I*	Voltage-Gated Calcium Channel Subunit Alpha 1I	Calcium signaling in neurons	NM_021096.3	22:40066855	c.4435C > T	p.Leu1479Phe	Not present	B	P	B	P	695

ExAC, frequency of the identified variants in the exome aggregation consortium. Four in silico prediction tools of functional mutation impact were used: ‘Sorting Tolerant From Intolerant’ (*SIFT*), ‘Polymorphism Phenotyping v2’ (*Polyphen2*); ‘Protein Variation Effect Analyzer’ (*PROVEAN*) and Mutation Taster2. The output results were classified as: likely pathogenic (*P*), likely benign (*B*) and not available (*NA*). Conservation scores refer to the conservation level of the nucleotide at the position of the identified variant between 46 species of vertebrates based on PhastCons. It ranges from 0 to 1000: the highest, the more conserved during evolution

**Table 6:** Clinical summary of patients with variants in new candidate disease genes

Proband	Gene variant	Age (years)	Onset of the signs	Microcephaly	Sitting alone	Ambulation	Respiratory function	Epilepsy	Hand use	Stereotypies	Language	Total score
3	*AGAP6*	14	3	1	2	4	1	1	3	2	2	19
8	*SCN1A, MGRN1, BTBD9*	7	3	1	1	4	1	2	1	3	2	18
11	GRIN2B, SEMA6B	3	2	0	1	3	0	1	1	1	2	10
12	*VASH2*	11	3	1	1	4	1	0	2	2	1	15
13	*CHRNA5*	10	3	1	2	4	1	1	2	3	2	19
14	ZNF620, GRAMD1A, NOC3L, GPATCH2	2	3	1	1	2	0	1	3	2	2	15
19	GABBR2, ATP8B1	2	3	1	1	4	0	0	3	2	2	16
20	*HAP1*	24	3	1	1	1	1	0	2	2	1	12
21	*PDLIM7, SRRM3*	5	3	1	3	4	0	1	2	1	2	17
22	*ANKRD31*	17	3	0	1	2	0	1	2	3	2	14
23	*CACNA1I*	1/8	3	1	1	2	1	0	3	3	2	16

Clinical scores of our series of patients according to Pineda scale. Severity classification ranges from 0 to 4 as follows: age of onset of first signs (1: >24 months; 2: 12–24 months; 3: 0–12 months), microcephaly (0: absent; 1: present), sitting alone (1: acquired < 8 months; 2: seat and mantains; 3: seat and lost), ambulation (0: acquired < 18 months, 1: acquired < 30 months; 2: acquired > 30 months; 3: lost acquisition; 4: never acquired), respiratory function (0: no dysfunction; 1: hyperventilation and/or apnea), epilepsy (0: absent; 1: present and controlled; 2: uncontrolled or early epilepsy), hands use (0: acquired and conserved; 1: acquired and partially conserved; 2: acquired and lost; 3: never acquired), onset of stereotypies (1: > 10 years, 2: > 36 months; 3: 18–36 months) and languages (0: preserved and propositive; 1: lost; 2: never acquired). The total score is the sum of the scores of each clinical feature

### Neurological phenotype of candidate genes in *C. elegans*

**Fig. 2:**
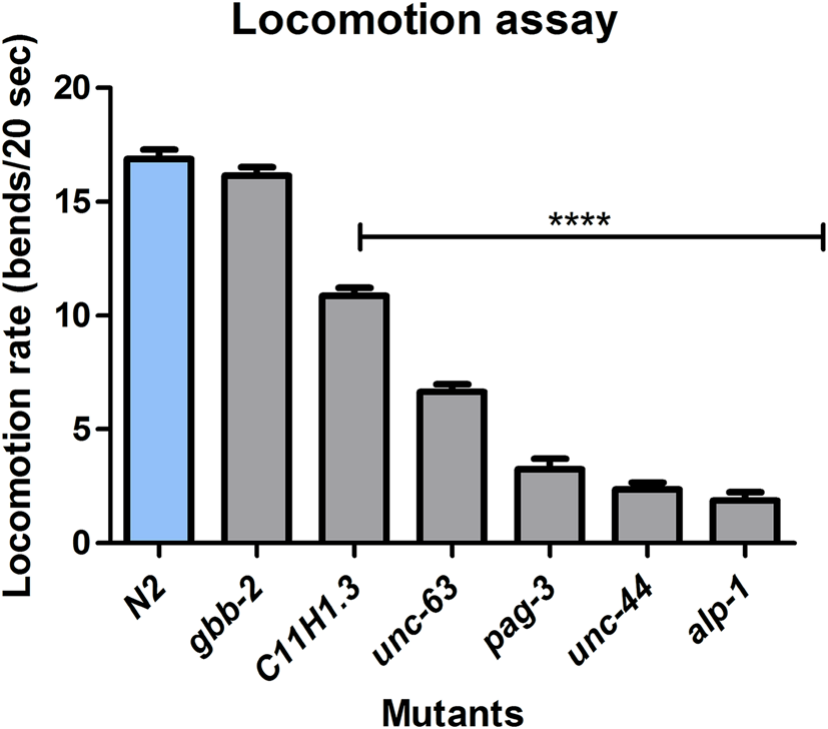
Locomotion assay in *Caenorhabditis elegans*. Functional validation of mutations was performed by measuring the locomotion rate, expressed in average of measuring, *in C. elegans*. Each mutant strain was compared to a wild-type N2 control strain by measuring worm body bends during 20 s in three independent sets of experiments. Locomotion rates of mutants, represented by *C11H1.3* (*MGRN1*), *unc*-*63* (*CHRNA5*), *pag*-*3* (*ZNF620*), *unc*-*44* (*ANKRD31*) and *alp*-*1* (*PDLIM7*) are significantly lower compared to that of the N2 control strain (*p* < 0.0001), on the contrary *gbb*-*2* (*GABBR2*) mutant move similarly. Standard error of the mean (SEM) values is shown. *p* values obtained according to Student’s *t* test. ***p < 0.0001

To demonstrate a neurological effect for a loss of function of the detected genes that had not previously been associated with neurodevelopmental disorders (Table 5), we used the model organism *C. elegans* to confirm the genotype-phenotype correlation. We obtained all the available *C. elegans* mutants that carry deleterious mutations in the orthologous genes to those human genes with potentially pathogenic mutations in the patients. In this model, backcrossing is a commonly used procedure to obtain a specific mutant strain without any secondary mutations from its genetic composition. Under these conditions, we were able to test six available mutant strains that were backcrossed at least three times to prove that any observed phenotype was really associated to specific mutations in the orthologous genes. To this end, we studied the *C. elegans* mutants carrying deleterious mutations in the gene orthologs of the human genes *PDLIM7*, *ANKRD31*, *ZNF620*, *CHRNA5*, *MGRN1* and *GABBR2* described in Table 7. Considering that the loss of normal movement and coordination is one of the clearest signs shown by Rett patients, we performed a locomotion assay of the nematodes as previously described (Sawin et al. 2000), using the wild-type N2 strain as a control (Supplementary Video 1). We observed that in 83.3 % (5 of 6) of the cases the mutation of the ortholog of the human exome sequencing identified genes in *C. elegans* exhibited a locomotion defective phenotype (Fig. 2). The most severe phenotypes were represented by *alp*-*1*, *unc*-*44* and *pag*-*3*, with mutations in the orthologs of PDZ and LIM domain protein 7 (*PDLIM7*), ankyrin repeat containing protein *ANKRD31* and the zinc protein *ZNF620*, respectively (Fig. 2 and Supplementary Videos 2, 3 and 4). The case of *alp-1* was particularly interesting, because mutant worms were not only thinner than usual and completely locomotion defective, but they exhibited transitory spasms. Significant defects, such as slower locomotion and uncoordinated movement, were also observed in the mutants of *unc*-*63* and *C11H1.3*, the *C. elegans* orthologs of the genes coding for the neuronal acetylcholine receptor subunit alpha-5 (*CHRNA5*) and mahogunin RING finger protein 1 (*MGRN1*), respectively. Although we did not find a clear locomotion defect in the *gbb*-*2* mutant (the ortholog of *GABBR2*) (Fig. 2), it occurs in the *gbb*-*1*;*gbb*-*2* double mutant (Dittman and Kaplan 2008), *gbb*-*1* being the *C. elegans* ortholog of *GABBR1* (gamma-aminobutyric acid type B receptor subunit 1). The clinical picture of the particular RTT cases with mutations in the genes studied in *C. elegans* is shown in Table 6.

**Table 7:** Phenotype in *Caenorhabditis elegans*

Human gene	Ortholog in C.elegans	Similarity (%)	Identity (%)	Mutation in C.elegans	Locomotion phenotype	Neurological phenotypes	Other phenotypes
*GABBR2*	*gbb*-*2*	53	34	Deletion	normal	Hypersensitivity to aldicarb	–
*MGRN1*	*C11H1.3*	58	41	Deletion	locomotion defective	–	–
*CHRNA5*	*unc*-*63*	58	40	Deletion	locomotion defective	Uncoordinated locomotion with strong levamisole resistance	–
*ZNF620*	*pag*-*3*	65	47	Deletion	locomotion defective	Altered neurosecretion and up-regulation of DCV (Dense Core Vesicles) components	–
*ANKRD31*	*unc*-*44*	59	39	Deletion	locomotion defective	Asymmetric dynamics of axonal and dendritic microtubules defects	–
*PDLIM7*	*alp*-*1*	65	47	Deletion	locomotion defective	–	Defects in actin filament organization in muscle cells

## Discussion

Our results indicate that the existence of de novo variants in genes with potential neurological functionalities, such as neuronal receptors (*GABBR2* and *CHRNA5*), axon guiders (*SEMA6B*), synaptic ionic channels (*CACNA1I*) and others, contribute to the development of RTT-like clinical phenotypes in the context of wild-type sequences for standard Rett genes such as *MECP2* and *FOXG1*. These patients share most of the clinicopathological features of classic RTT syndrome, such as stereotypic hand movements, relative microcephaly, and onset of the disease after the age of 12 months. Thus, exome sequencing is a powerful tool for genetically characterizing these enigmatic cases. In this regard, once a new candidate gene has been identified, it is now possible to design specific sequencing strategies for the molecular screening of this particular target in larger populations of patients with intellectual disability. The strategy based on exome sequencing patients who have RTT features, but no known mutations in the usual genes, has recently been used in other smaller series of patients (Grillo et al. 2013; Okamoto et al. 2015; Hara et al. 2015; Olson et al. 2015; Lopes et al. 2016). Most importantly, our study and the aforementioned previous reports strengthen the concept that a mutational heterogeneous profile hitting shared neurological signaling pathways contributes to RTT-like syndromes. Examples of confluence in the same molecular crossroads include the gamma-aminobutyric type B receptor subunit 2 (*GABBR2*) de novo variant, described here, and the formerly identified variant in the gamma-aminobutyric acid receptor delta gene (*GABRD*) (Hara et al. 2015). Interestingly, a second RTT-like patient has been identified as being a carrier of a de novo *GABBR2* variant (Lopes et al. 2016), highlighting the likelihood that this gene and pathway contribute to the clinical entity. Another example of similarly targeted genes in RTT-like patients is that of the proteins containing ankyrin-repeats that are involved in postsynaptic density (Durand et al. 2007). This study has revealed de novo variants in the ankyrin repeat containing proteins *AGAP6* and *ANKRD31* in RTT-like patients, and the presence of de novo variant of the SH3 and multiple ankyrin repeat domain3 protein (*SHANK3*) (Hara et al. 2015) and ankyrin-3 (*ANK3*) (Grillo et al. 2013) has been reported in two RTT-like patients. A final example of the convergence of cellular pathways to provide a common RTT-like phenotype is represented by the disruption of the ionic channels. We found the existence of a voltage-gated calcium channel subunit alpha 11 (*CANA1I*) de novo variant in an RTT-like patient. Additionally, the presence of de novo variants in the calcium release channel *RYR1* (Grillo et al. 2013) and the sodium voltage-gated channel alpha subunit 2 (*SCN2A*) (Baasch et al. 2014) in two other RTT-like probands have been reported. It is also intriguing that in our study a variant in HAP was found, whereas in similar series heterozygous variants in huntingtin (HTT) have been described (Lopes et al. 2016; Rodan et al. 2016), further reinforcing the links between Huntington’s disease and Rett syndrome (Roux et al. 2012). Another interesting case is provided by TCF4, which is associated with Pitt–Hopkins syndrome (Sweatt 2013), where in addition to our study, others have found mutations in RTT-like patients (Lopes et al. 2016). This observation could be of interest for clinicians due to phenotypic similitudes such as intellectual disability, stereotypic movement, apneas and seizures (Marangi et al. 2012).

Our findings also suggest that a substantial degree of clinical overlap can exist between the features associated with RTT and those of other neurodevelopmental disorders. Our exome sequencing effort indicated that probands originally diagnosed as RTT-like patients were, in fact, carriers of well-known pathogenic de novo mutations linked to Dravet Syndrome (*SCN1A*), myoclonic-atonic epilepsy (*SCLC6A1*), or early infantile epileptic encephalopathies 24 (*HCN1*) and 27 (*GRIN2B*). The purely clinical classification of these patients, without a thorough genetic study, can be difficult because some of these patients are composites that carry at least two pathogenic variants. For example, in our cases, the Dravet syndrome patient also had a de novo variant in *BTBD9* associated with the development of restless leg syndrome. In addition, among the newly identified candidate genes associated with RTT-like features, a few of these patients simultaneously carried two de novo variants (e.g., probands 8, 19 and 21), further complicating the tasks of correctly diagnosing and managing these individuals.

Finally, the studies performed in *C. elegans* validate the functional relevance for nervous system function of the newly proposed candidate genes. Future studies would be necessary to assess the role of the specific variants identified, such as rescuing the defects with the expression of normal cDNAs versus cDNAs containing the mutation, ideally using cDNAs of human origin to prove similar function of the gene in the two species. It is also relevant to mention that for some of the newly reported mutated genes in our RTT-like patients, there are mice models targeting the described loci that show neurological phenotypes such as BTBD9 (motor restlessness and sleep disturbances) (DeAndrade et al. 2012), MGRN1 (spongiform neurodegeneration) (He et al. 2003), SEMA6B (aberrant mossy fibers) (Tawarayama et al. 2010), CHRNA5 (alterations in the habenulo-interpeduncular pathway) (Fowler et al. 2011), GABBR2 (anxiety and depression-related behavior) (Mombereau et al. 2005) and HAP1 (depressive-like behavior and reduced hippocampal neurogenesis) (Chan et al. 2002; Xiang et al. 2015).

## Conclusions

Overall, this study demonstrates the genetic mutational diversity that underlies the clinical diagnosis of patients with clinical features that resemble RTT cases. Once the recognized *MECP2*, *CDKL5* and *FOXG1* mutations have been discarded, exome sequencing emerges as a very useful strategy for the more accurate classification of these patients. The de novo variants identified by this approach can modify the first diagnostic orientation towards another neurodevelopmental disorder, or pinpoint new genes involved in the onset of RTT-like features. Interestingly, most of these new targets are involved in the same functional networks associated with correct neuronal functionality. Further research is required to understand the role of these proteins in the occurrence of neurodevelopmental diseases. Additional functional experiments, such as the *C. elegans* assays used in this study, would be extremely helpful for this purpose.

## Electronic supplementary material

Below is the link to the electronic supplementary material.

Supplementary material 1 (DOCX 19 kb)

Supplementary material 2 (DOCX 16 kb)

Supplementary material 3 (MOV 1409 kb)

Supplementary material 4 (MOV 590 kb)

Supplementary material 5 (MOV 657 kb)

Supplementary material 6 (MOV 3103 kb)
