# Supplementary material for: Whole exome sequencing of Rett syndrome-like patients reveals the mutational diversity of the clinical phenotype
Source: Hum Genet. 2016 Aug 19;135(12):1343–54. doi: 10.1007/s00439-016-1721-3 (PMC5065581; doi:10.1007/s00439-016-1721-3)
Supplement: Supplementary file 1 — Supplementary material 1 (DOCX 19 kb) [file 439_2016_1721_MOESM1_ESM.docx]

| **Supplementary Table 1** Technical statistics of WES. Overall coverage statistics for each individual of the families, considering the regions captures using Exome Enrichment Kit, and number of reads in the position of the variation. | | | | |
| --- | --- | --- | --- | --- |
| **Gene** | **IDs** | **Average of coverage** | **Numer of reads** | **Variant: genomic coordinates** |
| *AGAP6* | **Patient 3** | 51,7222 | 30 | 10:51748528 |
|  | Mother | 19,1904 | 59 |  |
|  | Father | 19,8947 | 49 |  |
| *HCN1* | **Patient 4** | 37,9988 | 33 | 5:45396665 |
|  | Mother | 21,2688 | 11 |  |
|  | Father | 53,6885 | 22 |  |
| *SCN1A* | **Patient 8** | 63,9124 | 98 | 2:166866266 |
|  | Mother | 55,4485 | 98 |  |
|  | Father | 66,9241 | 91 |  |
| *MGRN1* | **Patient 8** | 63,9124 | 4 | 16:4723583 |
|  | Mother | 55,4485 | 1 |  |
|  | Father | 66,9241 | 3 |  |
| *BTBD9* | **Patient 8** | 63,9124 | 100 | 6:38256093 |
|  | Mother | 55,4485 | 101 |  |
|  | Father | 66,9241 | 105 |  |
| *TCF4* | **Patient 10** | 57,353 | 44 | 18:52901827 |
|  | Mother | 45,2247 | 87 |  |
|  | Father | 58,4836 | 77 |  |
| *SEMA6B* | **Patient 11** | 38,1451 | 21 | 19:4555540 |
|  | Mother | 43,3847 | 16 |  |
|  | Father | 31,635 | 31 |  |
| *GRIN2B* | **Patient 11** | 38,1451 | 45 | 12:13764782 |
|  | Mother | 43,3847 | 44 |  |
|  | Father | 31,635 | 41 |  |
| *VASH2* | **Patient 12** | 56,4905 | 84 | 1:213161902 |
|  | Mother | 68,9685 | 84 |  |
|  | Father | 63,1402 | 109 |  |
| *CHRNA5* | **Patient 13** | 63,6187 | 53 | 15:78882481 |
|  | Mother | 65,3851 | 64 |  |
|  | Father | 58,9521 | 67 |  |
| *ZNF620* | **Patient 14** | 55,267 | 27 | 3:40557941 |
|  | Mother | 57,7491 | 34 |  |
|  | Father | 60,002 | 28 |  |
| *GRAMD1A* | **Patient 14** | 55,267 | 56 | 19:35506764 |
|  | Mother | 57,7491 | 42 |  |
|  | Father | 60,002 | 43 |  |
| *NOC3L* | **Patient 14** | 55,267 | 26 | 10:96097586 |
|  | Mother | 57,7491 | 52 |  |
|  | Father | 60,002 | 36 |  |
| *GPATCH2* | **Patient 14** | 55,267 | 96 | 1:217784371 |
|  | Mother | 57,7491 | 111 |  |
|  | Father | 60,002 | 104 |  |
| *SLC6A1* | **Patient 17** | 62,2434 | 43 | 3:11067528 |
|  | Mother | 56,8694 | 53 |  |
|  | Father | 61,0401 | 66 |  |
| *GABBR2* | **Patient 19** | 67,6492 | 46 | 9:101133817 |
|  | Mother | 35,6295 | 30 |  |
|  | Father | 65,7731 | 65 |  |
| *ATP8B1* | **Patient 19** | 67,6492 | 61 | 18:55328507 |
|  | Mother | 35,6295 | 38 |  |
|  | Father | 65,7731 | 70 |  |
| *HAP1* | **Patient 20** | 57,3956 | 11 | 17:39890655 |
|  | Mother | 63,086 | 19 |  |
|  | Father | 52,3069 | 15 |  |
| *PDLIM7* | **Patient 21** | 63,5277 | 56 | 5:176910933 |
|  | Mother | 59,2951 | 28 |  |
|  | Father | 58,6697 | 20 |  |
| *SRRM3* | **Patient 21** | 63,5277 | 52 | 7:75890878 |
|  | Mother | 59,2951 | 59 |  |
|  | Father | 58,6697 | 53 |  |
| *ANKRD31* | **Patient 22** | 58,6697 | 75 | 5:74518166 |
|  | Mother | 56,1542 | 66 |  |
|  | Father | 57,2364 | 65 |  |
| *CACNA1I* | **Patient 23** | 20,1538 | 32 | 22:40066855 |
|  | Mother | 9,48446 | 9 |  |
|  | Father | 56,6495 | 75 |  |
