# Supplementary material for: Whole exome sequencing of Rett syndrome-like patients reveals the mutational diversity of the clinical phenotype
Source: Hum Genet. 2016 Aug 19;135(12):1343–54. doi: 10.1007/s00439-016-1721-3 (PMC5065581; doi:10.1007/s00439-016-1721-3)
Supplement: Supplementary file 2 — Supplementary material 2 (DOCX 16 kb) [file 439_2016_1721_MOESM2_ESM.docx]

| **Supplementary Table 2 Primers used for Sanger sequencing** | | |  |
| --- | --- | --- | --- |
| **Gene** | **Primers** | **Sequence** | **Genomic position (GRCh37/hg19)** |
| *AGAP6* | F | AGCGGGAAGACCATCTCTG | chr10:51748364-51748795 |
|  | R | GAAAGGAGCTCGAAGTGTGG |  |
| *HCN1* | F | CAGCAGACTGTTTCCACTTCA | chr5:45396198-45397021 |
|  | R | CATGCACAATAGCTGCCTGT |  |
| *SCN1A* | F | TTTTGTGTGTGCAGGTTTCATT | chr2:166866053-166866365 |
|  | R | AGGCCTATTTCTCTTGCATATCA |  |
| *MGRN1* | F | CTGGATTTGAGCCTGGTGAT | chr16:4723322+4723803 |
|  | R | CCCACGTTCAGCACAGACTA |  |
| *BTBD9* | F | TCCTGATGCCAAATCTTGTT | chr6:38255873-38256270 |
|  | R | GCACGCTATATCTCGTTGTTG |  |
| *TCF4* | F | TCAGCGCCCTCTAGTGAAAC | chr18:52901609-52902050 |
|  | R | TTAGCGGGCGAAGTTCTAAA |  |
| *SEMA6B* | F | TGGCCAAGGTCACACAGTAA | chr19:4555127-4555699 |
|  | R | AAGGCAGGCAAGAGATGAG |  |
| *GRIN2B* | F | TACAATCTAACCTAGGCCCTGG | chr12:13764558-13764935 |
|  | R | TGGATATGCTAGGGAAAATGCAG |  |
| *VASH2* | F | GCAAGGTTCAAGAGTACTGGGT | chr1:213161536+213162249 |
|  | R | TGGTGAGGCATAATGTTCAAAGC |  |
| *CHRNA5* | F | GAGCAGGGTCCCTATGTAGC | chr15:78881983+78882780 |
|  | R | CGCCATGGCATTATGTGTTGA |  |
| *ZNF620* | F | TAGCGTCAGCACACAACTCA | chr3:40557616+40558235 |
|  | R | GCTGGTGCTGAATCAGGGT |  |
| *GRAMD1A* | F | CTCACCCCTGAACCAATTGC | chr19:35506400+35507085 |
|  | R | AGAAGGAGAAACTGAGGCACA |  |
| *NOC3L* | F | TGTAGAAAATAGAAGTGGCAGGT | chr10:96097185-96097950 |
|  | R | CACATGAAGCACCTATAGCCA |  |
| *GPATCH2* | F | TGCTGGCAGTTCTTAGAGTCT | chr1:217784024-217784774 |
|  | R | TCAATGAGCCTAGCAAGAAAGC |  |
| *SLC6A1* | F | CTGTCTGACTCCGAGGTGAG | chr3:11067255+11067936 |
|  | R | GACGATGATGGAGTCCCTGA |  |
| *GABBR2* | F | GTGACCTGGGTCTGGTAAGTG | chr9:101133405-101134056 |
|  | R | TTCCTTTGACAAGGTCCCCAG |  |
| *ATP8B1* | F | AATCTTGGGAATGGTACTCCTGG | chr18:55328163-55328763 |
|  | R | ACCTTATTTTCCTCTTCGCATCC |  |
| *HAP1* | F | CATCCGGAACTTGCACTCG | chr17:39890418-39891088 |
|  | R | CTTCCTCCAGCTCCCGAATA |  |
| *PDLIM7* | F | GTGTTCCCGTGACCCAGG | chr5:176910565-176911259 |
|  | R | AGCCCTACCCAGAAATGCAG |  |
| *SRRM3* | F | GTCACTCTGTACAAGGGACCT | chr7:75890525+75891203 |
|  | R | CTGCTTGTCTAACTGGCACC |  |
| *ANKRD31* | F | GGACGCATCAATAGGTGCAG | chr5:74517744-74518408 |
|  | R | GCTTCCAGTCAACAGTAGGC |  |
| *CACNA1I* | F | CCACTGCCAACCTGAGTGA | chr22:40066439+40067235 |
|  | R | CACAGTCATTGCCACCCATG |  |

F, Forward; R, Reverse.
